# Supplementary material for: Tolerability and Efficacy of s.c. IgG Self-Treatment in ME/CFS Patients with IgG/IgG Subclass Deficiency: A Proof-of-Concept Study
Source: J Clin Med. 2021 May 29;10(11):2420. doi: 10.3390/jcm10112420 (PMC8198960; doi:10.3390/jcm10112420)
Supplement: Supplementary file 1 [file jcm-10-02420-s001.zip › S1_clinical study protocol.pdf]

Supplementary Data

S1: Trial study protocol

Tolerability and efficacy of s.c. IgG self-treatment in patients with ME/CFS: a proof-of-concept study

Carmen Scheibenbogen[*1,2], Franziska Sotzny[1], Jelka Hartwig[1], Sandra Bauer[1], Helma Freitag[1], Kirsten Wittke[1], Wolfram Doehner[2,3], Nadja Scherbakov[2,3], Madlen Loebel[4], Patricia Grabowski[1]

Journal of Clinical Medicine, 2021

[*] **Corresponding author:**

Carmen Scheibenbogen
Institute of Medical Immunology, Charité – Universitätsmedizin Berlin, corporate member of Freie Universität Berlin and Humboldt-Universität zu Berlin, Germany

Carmen.Scheibenbogen@charite.de

13.12.16

CLINICAL STUDY PROTOCOL

PROOF OF CONCEPT STUDY OF HYQVIA IN PATIENTS WITH IMMUNOGLOBULIN DEFICIENCY AND RECURRENT INFECTIONS WITH CHRONIC FATIGUE SYNDROME

Sponsor:
Charité Universitätsmedizin Berlin

Deputy
Prof. Dr. med. Carmen Scheibenbogen
Institut für Med. Immunologie

Charité Universitätsmedizin Berlin

Principal investigator:

PD Dr. med. Patricia Grabowski

Institut für Med. Immunologie

Charité Universitätsmedizin Berlin

Prüfplan-Code: IMI-2016-2

EudraCT-Nummer: 2016-002370-12

Version 2.0

Die Informationen in diesem Prüfplan sind streng vertraulich zu behandeln. Sie dienen nur zur Information des Sponsors, der Prüfer, der Studienmitarbeiter, der Ethikkommission, der Behörden und der Patienten. Dieser Prüfplan darf ohne Zustimmung des Sponsors oder des Leiters der klinischen

Prüfung (LKP) nicht an Dritte weitergegeben werden.

PD Dr. med. Patricia Grabowski
(Principal investigator, LKP,
Leiterin der klinischen Prüfung)

Unterschrift Datum

Prof. Dr. med. Carmen Scheibenbogen,

Charité Universitätsmedizin Berlin
Sponsor, Deputy

Unterschrift Datum

I. Synopsis

Sponsor<br>Deputy	Charité Universitätsmedizin Berlin<br>Prof. Dr. med. Carmen Scheibenbogen<br>Institut für Med. Immunologie, Charité
Principal investigator	PD Dr. med. Patricia Grabowski<br>Institut für Med. Immunologie, Charité
Title:	Proof of concept study of HyQvia in patients with immunoglobulin deficiency and recurrent infections with chronic fatigue syndrome
Indication:	Immunoglobulin deficiency and recurrent infections with chronic fatigue syndrome
Phase:	II
Study type:	monocenter proof of concept study
Study design:	prospective, open-label, non-controlled
Patients:	15

Objectives:	Primary Objective: to assess<br>• efficacy to improve fatigue <u>and physical functioning</u><br>Secondary Objectives: to assess<br>• number and severity of infections<br>• tolerability<br>• identify marker for response
Criteria for evaluation:	Efficacy:<br>• questionnaires assessing severity of fatigue and disease-related symptoms<br>• functional assessment of physical and mental fatigue<br>• rate, type and severity of infections<br>Safety:<br>• Adverse events (according to Common Toxicity Criteria [CTC])

Inclusion and exclusion criteria:	Inclusion criteria (all must be fulfilled):<br>• Patients of both genders aged 18 -65 years<br>• immunoglobulin deficiency (Common Variable Immunodeficiency (CVID), IgG deficiency, IgG subclass deficiency)<br>• no previous IgG treatment<br>• serious bacterial infection or other recurrent infections: > 4 infections during the last year prior to inclusion<br>• Chronic Fatigue Syndrome (CFS) with $\leq$ 50 points on Bell scale<br>• Normal ALAT, ASAT, AP, g-GT, PTT and INR/Quick, serum creatinine, normal or slightly reduced haemoglobin, leucocytes, thrombocytes<br>• Signed informed consent.<br><br>Exclusion criteria:<br>• Concomitant anti-coagulation therapy or history of thrombotic episodes (including deep vein thrombosis, myocardial infarction, cerebrovascular accident, pulmonary embolism) within 12 months prior to screening or a history of thrombophilia<br>• IgA deficiency (IgA less than 0.07g/l) and known anti-IgA antibodies<br>• Persistent severe neutropenia (defined as an absolute neutrophil count (ANC<500/mm^3)<br>• History of or is positive at screening for one or more of the following: hepatitis B surface antigen (HBsAg), polymerase chain reaction (PCR) for hepatitis C virus (HCV), PCR for human immunodeficiency virus (HIV) Type I/II<br>• Subject is on preventative (prophylactic) systemic antibacterial antibiotics (at doses sufficient to treat or prevent bacterial infections)

- Subject is scheduled to participate in another interventional clinical study involving an investigational product or device during the course of the study
- Subject has known hypersensitivity to any of the components of HyQvia
- Subject has severe dermatitis that would preclude adequate sites for safe product administration
- Subject is a family member or employee of the investigator
- Subject is dependent from sponsor
- Subject is not capable to understand questionnaires due to language or cognitive problems
- Subjects housed in an institution due to governmental or judicial authorities

Dosage and application: HyQvia 0.2-0.8g/kg body weight/month, subcutaneous

Treatment duration: Every patient will be treated for 12 months.

First patient first visit, FPFV:	1.1.2017
Last patient first visit, (LPFV):	1.6.2017
End of study last patient (LPLV):	30.9.2018
Final Report:	30.12.2018

Statistician: Dr. Jens Klotsche, GWT-TUD GmbH, Dresden

Statistical methods:	Descriptive methods. Intent-to-treat principle. In case of missing observations, last observation carried forward method.
GCP compliance:	This study will be conducted according the most recent version of the study protocol and the internationally accepted guidelines on Good Clinical Practice (ICH-GCP).
Funding:	The study is supported by an industry grant from Baxalta Healthcare Corporation, One Baxalta Parkway, Deerfield, Illinois 60015, USA. The company is represented in Germany by Baxalta Deutschland GmbH, Edisonstraße 4, 85716 Unterschleißheim.

II. Inhaltsverzeichnis

I. Synopsis 5

II. Inhaltsverzeichnis 10

II.a) Table listing 14

II.b) Figure listing 14

III. Abbreviations 15

1. Introduction 16

1.1. Study Rationale 17

1.2. Primary objective 17

1.3. Secondary objectives 17

2. Organisationsstruktur 18

2.1. Sponsor 18

2.2. Statistics 18

2.3. Study center and principle investigator 19

2.4. Funding 19

3. Study design and endpoints 20

3.1. Study design 20

3.1.1. Study schedule 20

3.1.2. Study visits 20

3.2. Study endpoints 22

3.2.1. Primary Endpoints 23

3.2.2. Secondary Endpoints 23

3.3. Discussion of study design 23

3.4. Study population 24

3.4.1. Inclusion criteria 24

3.4.2. Exclusion criteria 25

3.5. Withdrawal rules 26

3.6. Premature study termination 27

3.6.1. Termination of entire study 27

3.7. Treatment 27

3.7.1. Study treatment with HyQvia 27

3.7.2. Study medication 29

3.7.2.1. Labeling of study medication 30

3.7.2.2. Storage of study medication 30

3.7.3. Drug accountability 30

3.7.4. Dosing of study medication 30

3.7.5. Dosing and treatment schedule of study medication 31

3.7.6. Blinding 33

3.7.7. Previous and concomitant medication 33

3.7.8. Treatment after completion of clinical study 33

3.8. Effectiveness and safety parameters 33

3.8.1. Measurement of effectiveness and safety parameters 33

3.8.1.1. Primary End Points 33

3.8.1.2. Secondary End Points 34

3.8.1.3. Safety analysis 35

3.8.2. Appropriateness of methods 35

3.8.3. Pharmacokinetic measurement 35

3.9. Monitoring 36

3.10. Documentation 36

3.10.1. Data collection and pseudonymisation 36

3.10.2. Subject completion/ discontinuation 36

3.10.3. Archiving 37

4. Ethical and regulatory aspects 38

4.1. Independent ethical committee 38

4.2. Ethical implementation 38

4.2.1. Legal provisions and guidelines 38

4.3. Approval and registration 38

4.4. Information and consent of study participants 39

4.5. Patient insurance 39

4.6. Data privacy 39

5. Statistical methods and sample size calculation 40

5.1. Statistical analysis plan 40

5.1.1. Description of patient sample 40

5.1.2. Analysis of the primary efficacy endpoint 40

5.1.3. Analysis of the secondary efficacy endpoints 41

5.1.4. Subgroup analyses 42

5.1.5. Interim analyses 42

5.2. Sample size calculation 42

6. Documentation of adverse events and adverse reactions 43

6.1. Adverse event 43

6.2. Adverse Drug Reaction 45

6.3. Serious Adverse Event 45

6.4. Unexpected Adverse Events 46

6.5. Suspected Unexpected Adverse Reaction 46

6.6. Documentation and follow up of adverse events 46

6.7. AE Severity 46

6.8. Causality of adverse event 47

6.9. Reporting of serious adverse event, and pregnancies 49

7. Data utilisation and publication 50

7.1. Study reports 50

7.2. Publication 50

8. Changes to the study protocol 51

9. References 52

Appendix Questionnaires and CRF 54

II.a) Table listing

Table 1: Schedule 20

Table 2: Visit schedule **Fehler! Textmarke nicht definiert.**

II.b) Figure listing

Figure 1: Study schedule 20

III. Abbreviations

Abbreviation	Explanation
AE	Adverse Event (Unerwünschtes Ereignis)
CFS	Chronic Fatigue Syndrome
CRF	Case Report Form (Erhebungsbögen)
EC	Ethics committee
IgG	Immunoglobulin G
IV	intravenous
GCP	Good Clinical Practice
LKP	Principal Coordinating Investigator (Leiter der Klinischen Prüfung)
PEI	Paul-Ehrlich-Institut
SAE	Serious Adverse Event
SBI	Serious bacterial infections
SC	Subcutaneous
SUSAR	Suspected Unexpected Serious Adverse Reaction

1. Introduction

Chronic Fatigue Syndrome (CFS) is a frequent and mostly severe disease with an estimated prevalence of 0.3% in Germany. The patients suffer from sustained exhaustion accompanied by various physical and mental symptoms. CFS onset is typically with an infection and many patients suffer from frequently recurrent infections. The underlying pathological mechanism in CFS is not known so far. However, there is ample evidence of dysregulation of the immune response, and both immune activation and deficiency can be found. A recent trial from Norway provided evidence that in a subset of CFS patients B cell depletion with rituximab is effective [1]. Further we could show presence of autoantibodies to the ß adrenergic and muscarinic acetylcholine receptor in a subgroup of 30% of patients with normalisation in responders to rituximab [2].

Previously, we analysed 468 patients with CFS who presented at our outpatient clinic for immunodeficiency. 25% of the CFS patients had decreased serum levels of at least one antibody class or subclass with immunoglobulin (IgG) subclass deficiency as most common phenotype. Patients with immunoglobulin deficiency had more frequently an increased rate of infections, mostly of the respiratory tract [3, 4].

Four randomized controlled clinical trials of intravenous (IV) immunoglobulin replacement therapy with monthly doses ranging from 0.5 – 2g/kg body weight can be found in PubMed showing inconsistent results with two positive and two negative studies. In these studies patients had not been selected for concomitant antibody deficiency [5].

Until recently there was little interest of academia and pharmaceutical companies into clinical trials in CFS presumably due to complexity of the disease and paucity of knowledge. In 2014 an Institute of Medicine committee produced a 300-page consensus report on CFS [6]. Based on this report, NIH announced a concerted research program in October 2015. Within the EU the COST action proposal EUROMENE (European Network on ME/CFS) was recently granted (Carmen Scheibenbogen is the German management committee representative and leads the biomarker group).

1.1. Study Rationale

Chronic fatigue syndrome (CFS) is a severe disease characterized by various symptoms of immune dysfunction and is frequently associated with immunoglobulin deficiency. IgG substitution may have therapeutic effect in CFS both by ameliorating susceptibility to infections and due to immunomodulatory effects of IgG.

1.2. Primary objective

The objective of this study is to improve chronic fatigue and physical functioning as assessed by Chalder Fatigue Scale and SF-36, physical function domain, respectively.

1.3. Secondary objectives

Secondary study objectives of this study are

- to assess the frequency and severity of infections
- to assess the tolerability of HyQvia in patients with CFS and patient satisfaction with handling of self IgG application and the mechanical pump
- to identify functional markers for response

2. Organisationsstruktur

2.1. Sponsor

Sponsor

Charité Universitätsmedizin Berlin
Deputy: Prof. Dr. med. Carmen Scheibenbogen
Institut für klinische Immunologie
Campus Virchow-Klinikum
Föhrer Str. 15 / Südstr. 2
13353 Berlin
t: +49 30 450 524 103
f: +49 30 450 7 524103

Leiter der klinischen Prüfung

PD Dr. med. Patricia Grabowski
Institut für Medizinische Immunologie
Campus Virchow-Klinikum
Föhrer Str. 15 / Südstr. 2
13353 Berlin
t: +49 30 450 624 103
f: +49 30 450 518 905

2.2. Statistics

Statistician: Dr. Jens Klotsche, GWT-TUD GmbH, Dresden

Project management: not applicable

Monitoring: Dr. Michael Teubner, GWT-TUD GmbH, Dresden

SAE management: PD Dr. med. Patricia Grabowski, Institut für Medizinische Immunologie

Scientific advice by: not applicable

2.3. Study center and principle investigator

The Study is conducted as monocenter study at the Institute für Medizinische Immunologie at the Charité. Principal Investigator is PD Dr. med. Patricia Grabowski.

2.4. Funding

The study is supported by an industry grant from Baxalta Healthcare Corporation, One Baxalta Parkway, Deerfield, Illinois 60015, USA. The company is represented in Germany by Baxalta Deutschland GmbH, Edisonstraße 4, 85716 Unterschleißheim.

3. Study design and endpoints

3.1. Study design

The study is a prospective, open-label, non-controlled, mono-center proof-of-concept study

3.1.1. Study schedule

Table 1: Schedule

First patient first visit, FPFV:	1.1.2017
Last patient first visit, (LPFV):	1.6.2017
End of study last patient (LPLV):	30.9.2018
Final Report:	30.12.2018

Figure 1: Study schedule

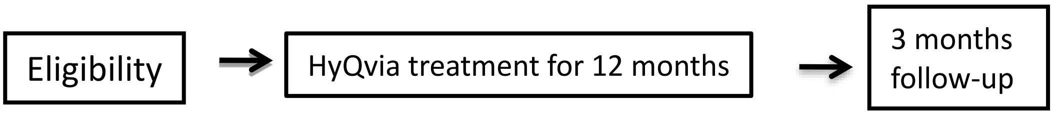

The following section provides the schedule of procedures and assessments for the various parts of the study.

3.1.2. Study visits

Six clinical visits are planned for this study. Study duration for an individual patient will be 15 months.

Table 2: Visit schedule

**Visit**	**0**	**1**	**2**	**3**	**4**	**5**	**6**
	Screening	Treatment	Treatment	Treatment	Treatment	Treatment	Follow up
Study day	0	1	90<br>Month 3	180<br>Month 6	270<br>Month 9	360<br>Month 12	450<br>Month 15
deviation	-28 days		±14 days	±14 days	±14 days	±14 days	±14 days

Procedures	Screening / enrollment visit	Follow-up visit(s) at months 3,6,9,12	Termination visit month 15
Informed Consent (mandatory)	X		
Eligible Patients Criteria (mandatory)	X		
**Documentation**			
**Demographic data:**			
Height, Weight	X	X	X
Year of birth	X		
**Medical history:**			
CFS onset, course and symptoms	X	X	X
Concomitant medication	X	X	X
Concomitant and incidental diseases	X	X	X
Family history	X		
**Immunoglobulin therapy:**			
Previous immunoglobulin therapy	X		
Tolerability of current study medication		X	X
**CFS assessment**			
Canadian Criteria and Bell score	X		X
Chalder Fatigue scale and SF-36 (primary aim)	X	X	X
CFS Symptomes Scale and COMPASS-31	X	X	X
Viofit activity tracker	X	X	X
ECHO and assessment of vessel function	X	X[1]	
Bioelectrical impedance analysis (BIA)	X	X[1]	
Assessment of arm and leg muscle power	X	X[1]	
Sitting and standing pulse and blood pressure	X	X	
Clinical assessment	X	X	X
**Infection assessment**	X	X	X
Infection history by physician	X	X	X
Infection diary by patient	X	X	X
**Laboratory tests**			
TSH, HbA1c, ferritin	X		
IgG/A/M, IgG subclasses	X	X	X
ALT, AST, AP, yGT	X	X	X
Serum creatinine, electrolytes	X	X	X
CBC	X	X	X
CrP, IL-8 in erythrocytes	X	X	X
B cell/B cell subsets	X	X[1]	X
EBV PCR	X	X[2],[1]	X[2]
EBV antibodies	X	-	-
Autoantibodies	X	X	X
		[1] only month 12<br>[2] only if positive pretreatment	

3.2. Study endpoints

3.2.1. Primary Endpoints

The primary endpoints refer to effectiveness regarding to improve fatigue and physical functioning assessed by the Chalder Fatigue Scale and the SF-36 physical domain, respectively at study inclusion and follow-up at 12 months (see 3.8.1).

3.2.2. Secondary Endpoints

- Effectiveness to control infections documented by patient’s diary (type of infection, symptoms, duration, severity, antibiotics, hospitalization).
- *Functional assessment of fatigue by measuring muscle strength, composition of body tissues by bioimpedance, vessel function by flow mediated dilatation and activity tracking.*
- Additional questionnaires to assess CFS symptoms and physical functioning: CFS symptom scoring, COMPASS-31, SF-36 physical functioning.
- Assessment of the tolerability of HyQvia in patients with CFS and patient satisfaction with handling of self IgG application and the mechanical pump.

3.3. Discussion of study design

This is an open-label, non-controlled proof of concept study. There is a medical need for patients with immunoglobulin deficiency and recurrent infections to obtain immunoglobulins, and HyQvia is approved for this indication. Evidence on the effectiveness of IgG in the treatment of CFS is limited [5]. Against this background, a small set of patients (n= 15), mainly based on feasibility considerations, will be investigated in this study. In case the HyQvia would be effective, defined as an improvement of fatigue and physical functioning (improved Chalder Fatigue Scale and SF-36 physical functioning score as described in the statistical analysis plan (5.1) this would warrant a consecutive randomized placebo-controlled trial.

Benefit-risk assessment

There is a medical need for patients with immunoglobulin deficiency and recurrent infections to obtain immunoglobulins, and HyQvia is approved for this indication [7, 8]. It is not known whether immunoglobulins such as HyQvia can improve CFS, but there are reports that IgG might be beneficial in this indication [5]. The study is not a typical interventional study, as the patients would receive the treatment (at least of the medication class) anyway, and only the questionnaires and diaries for the assessment of CSF and fatigue could impose extra effort to the patients. Overall, the participation in the study poses does not alter the benefit-risk assessment for the patient compared to the situation where the same agent would be prescribed for the same indication outside the context of a study.

3.4. Study population

Male or female adult patients with Chronic Fatigue Syndrome (CFS) with concomitant immunoglobulin deficiency and recurrent infections years with performance status according to Bell scale value of 50 or less.

At the outpatient Clinic for Adult Immunodeficiencies of the Department of Medical Immunology at the Charité, about 300 patients with CFS are seen per year, of whom about 25% have concomitant immunoglobulin deficiency nearly halve of patients with CFS (42%) suffer from recurrent infections [3, 4]. Based on experience we expect to see 15 patients eligible for the study during a 4-month inclusion period.

The distribution of male to female is approximately 1:2 in our patient cohort. Thus, we expect a similar distribution of patients in our study.

3.4.1. Inclusion criteria

- Age 18 -65 years, both genders
- immunoglobulin deficiency (Common Variable Immunodeficiency (CVID), IgG deficiency, IgG subclass deficiency)
- no previous IgG treatment

- serious bacterial infection or other recurrent infections: > 4 infections during the last year prior to inclusion, other than common cold
- CFS (performance status < 50 points on the Bell CFS scale)
- ALAT, ASAT, AP, gamma -glutamyl transpeptidase (g-GT) each < 3 x upper limit of normal (ULN)
- Normal or slightly reduced haemoglobin (>12 g/dl male, >11g/dl female), normal or slightly reduced leucocytes (>3/nl), normal thrombocytes (150 - 370/nl)
- Normal PTT and INR/Quick
- normal renal function: serum creatinine < 1.2 mg/dl
- Signed informed consent.

3.4.2. Exclusion criteria

- Subject is receiving anti-coagulation therapy or has a history of thrombotic episodes (including deep vein thrombosis, myocardial infarction, cerebrovascular accident, pulmonary embolism) within 12 months prior to screening or a history of thrombophilia
- Subject has IgA deficiency (IgA less than 0.07g/l) and known anti-IgA antibodies
- Subject has persistent severe neutropenia (defined as an absolute neutrophil count (ANC<500/mm^3)
- Subject has known history of or is positive at screening for one or more of the following: hepatitis B surface antigen (HBsAg), polymerase chain reaction (PCR) for hepatitis C virus (HCV), PCR for human immunodeficiency virus (HIV) Type I/II
- Subject is on preventative (prophylactic) systemic antibacterial antibiotics (at doses sufficient to treat or prevent bacterial infections)
- Other serious underlying medical conditions which could impair the ability of the patient to participate in the study
- Women of childbearing potential meeting any one of the following criteria

  - subject presents with a positive pregnancy test
  - subject is breast feeding
  - subject intends to begin nursing during the course of the study
  - subject is not willing to perform safe contraception (pearl-index <1) during the study participation
- Subject has participated in another clinical study and has been exposed to an investigational product (IP) or device within 30 days prior to study enrollment (exception: treatment with immunoglobulin pre-study)
- Subject is scheduled to participate in another interventional clinical study involving an IP or device during the course of the study
- Subject has known hypersensitivity to any of the components of the medicinal product
- Severe dermatitis that would preclude adequate sites for safe product administration
- Subject is a family member or employee of the investigator or sponsor
- Subjects who are housed in an institution due to governmental or judicial authorities

3.5. Withdrawal rules

In case if patients have severe side-effects to treatment (CTC grade 3 or 4, e.g. allergic response) they will be withdrawn from the study. The investigator and sponsor have the obligation to discontinue any subject from the study if, in their judgement, continued participation would pose an unacceptable risk for subject.

Any subject may voluntarily withdraw consent for continued participation and data collection at any time after consent without stating reasons.

Patients discontinued for safety reasons should be evaluated in ITT safety population.

In the statistical analysis, such patients will be evaluated as not treated per protocol. Patients who will be excluded will be substituted in case they received less than 3 months treatment. After withdrawal, patients will be treated by standard care, i.e. received symptomatic treatment.

3.6. Premature study termination

3.6.1. Termination of entire study

The study has to be terminated prematurely due to relevant medical or ethical reasons or lack of feasibility. Reasons for termination have to be documented in detail. Patients who are still on medication at the time of termination should get a final examination which is documented in the CRF.

Reasons for premature study termination are:

- Benefit-risk ratio has changed significantly for the patients (e.g. after availability of new evidence on HyQvia and/or CFS)
- Application of study medication is no longer justifiable.
- The clinical study is no longer feasible.

3.7. Treatment

3.7.1. Study treatment with HyQvia

Patients enrolled in the study are suffering from an immunoglobulin deficiency with a history of recurrent infection. Approved standard treatment is the substitution of immunoglobulins by intravenous or subcutaneous infusion of human IgG preparation. The dose required to achieve a sufficient level of IgG to protect patients from severe of frequent recurring infections is of the order of 0.4-0.8 g per kg body weight per month.

HyQvia is a product consisting of human normal immunoglobulin (IgG, 10%) and recombinant human hyaluronidase (rHuPH20, Hylenex®). The subcutaneous IgG is a 10%

solution that is prepared from human plasma consisting of at least 98% IgG, which contains a broad spectrum of antibodies (Kiovig®).

Recombinant hyaluronidase is an established therapeutic principle to facilitate the infusion of large volumes of fluids subcutaneously (e.g. Rituximab, Infliximab, Trastuzumab).

The two components are packaged together as a dual vial unit: IgG provides the therapeutic effect and the recombinant human hyaluronidase facilitates the dispersion and absorption of the IgG, resulting in a temporary increase in the permeability of the interstitial matrix that facilitates more rapid dispersion and absorption and improved bioavailability of the IgG 10% component.

HyQvia is indicated as replacement therapy in adults (i.e., aged 18 years or older) in primary immunodeficiency syndromes and in myeloma or chronic lymphocytic leukaemia with severe secondary hypogammaglobulinaemia and recurrent infections.

HyQvia was investigated in one main study lasting over a year and involving 87 patients with primary Immunodeficiency who had already had treatment with human normal IgG for at least three months [9]. The main measure of effectiveness was the number of serious bacterial infections that developed over one year of treatment. The study showed that HyQvia was able to reduce this number to 0.03 per year. This was below the predefined number needed to show effectiveness (which is one infection per year), and was similar to that seen with other licensed human normal immunoglobulin products.

Nearly all of the patients were able to attain the same dose interval with HyQvia as they had for IV administration. 94% of patients attained the same 3- or 4-week dosing whereas one decreased from 4 to 3 weeks, one from 4 to 2 weeks and one from 3 to 2 weeks (2 patients withdrew during the titration period).

Under SC HyQvia compared to IgG products, similar trough levels, relevant antibody levels, and effectiveness is seen, but the adverse event rate is reduced. Infusion duration and frequency are the same as for IgG products. There are no preclinical data that suggest an increased risk of mutagenicity, teratogenicity, fertility or neuronal development

HyQvia is marketed in the European Union since July 2013.

3.7.2. Study medication

HyQvia is a dual vial unit consisting of one vial of human normal immunoglobulin (Immune Globulin 10% or IgG 10%) and one vial of recombinant human hyaluronidase (rHuPH20). One ml of human normal immunoglobulin contains 100 mg of human normal immunoglobulin (purity of at least 98% immunoglobulin G (IgG). IgG 10% is a clear or slightly opalescent and colourless or pale yellow solution.

Trade name:	HyQvia
Substance:	human normal immunoglobulin 10% (IgG 10%)
ATC code:	J06BA
Formulation:	Solution for infusion (infusion)
Dosing:	0.2-0.8g/kg body weight / month
Supplier:	Baxalta Healthcare Corporation

The study drug HyQvia is approved in Europe since June 2013 as replacement therapy in adults (> 18 years) in primary immunodeficiency syndromes such as:

- congenital agammaglobulinaemia and hypogammaglobulinaemia
- common variable immunodeficiency
- severe combined immunodeficiency
- IgG subclass deficiencies with recurrent infections.

Further, HyQvia is also indicated as replacement therapy in adults (> 18 years) with severe secondary (= acquired) hypogammaglobulinaemia and recurrent infections in the indications myeloma or chronic lymphocytic leukaemia.

HyQvia should not be used in patients with:

- Hypersensitivity to the active substances or to any of the excipients

- Hypersensitivity to human immunoglobulins, especially in very rare cases of IgA deficiency when the patient has antibodies against IgA.
- Systemic hypersensitivity to hyaluronidase or recombinant human hyaluronidase.

HyQvia must not be given intravenously.

3.7.2.1. Labeling of study medication

Study medication HyQvia will be used in in the approved and commercially available packaging. Boxes will be labeled with a sticker „Zur Klinischen Prüfung." As the study is open label with a single treatment, neutral packaging is not required.

3.7.2.2. Storage of study medication

Study medication will be stored at 2-8°C in the Ambulanz für Immundefekte Erwachsener, Mittelallee 11, Charité, Campus Virchow Klinikum. Temperature of the refrigerator will be continuously recorded.

3.7.3. Drug accountability

The required amount of study medication for home self-treatment is given to the patient at every study visit.

Quantity and lot number are documented in the CRF. Patients will be advised to return used and unused medication at every visit. Unused medication will be destroyed.

3.7.4. Dosing of study medication

HyQvia will be given every 2 – 4 weeks via s.c. infusion per pump. The first injection will be given at the Immundefekt Ambulanz and patients will be trained for self therapy. The next injection(s) will either be given at the Immundefekt Ambulanz or as home therapy under supervision of a home care nurse trained in HyQvia treatment until the patient is reliably able to perform the treatment by him/herself. Further treatment will then be given by the patients as self therapy. In case the patient requires assistance he/she will either come to the

Ambulanz or a nurse will be sent to assist the patient with home treatment. In case of home treatment
a person should be present, who is capable of treating side effects or if severe side effects occur will call for medical help. Patients in home treatment and their caregivers should be trained on the early signs of hypersensitivity reactions.

The following doses will be given:

- Month 1, day 0: total 0.2g/kg body weight per month (one injection)
- Month 2: total 0.4g/kg body weight per month (given as bi-weekly or monthly injections)
- Months 3-12: total 0.8g/kg body weight per month (given as bi-weekly or monthly injections)

The dose will be escalated from months 1 to 3 and initially IgG is given at a slow infusion rate to ensure good tolerability (see 4.6.5). The amount of IgG needed to control infections is variable from patient to patients, ranging usually between 0.4 – 0.8g/kg body weight, and can often not be judged before patients have been on treatment for 6 or 12 months. Therefore all patients will receive the dose of 0.8g/kg/month to ensure that they receive a sufficient dose of IgG.

3.7.5. Dosing and treatment schedule of study medication

Dosing and treatment schedule will be applied according to approved Summary of Product Characteristics (SmPc; in German: Fachinformation HyQvia, Juni 2016).

The target dose of IgG 10% (HyQvia) for each patient is 0.8g/kg body weight per month subcutaneous.

In the first month of treatment one dose of 0.2g/kg/month will be given, in the 2nd month a single or 2 bi-weekly doses of total 0.4g/kg/month, from the 3rd month a single or 2 bi-weekly doses of total 0.8g/kg/month.

Administration of HyQvia will be exactly as described in the SmPC (Fachinformation):

The two components of the medicinal product must be administered sequentially through the same needle beginning with the recombinant human hyaluronidase followed by IgG 10%, as described below.

The HyQvia components may be infused using a variable rate mechanical pump with a subcutaneous needle set that is at least 24 gauge and an administration set that is compatible with the pump.

It is recommended that the recombinant human hyaluronidase component be administered at a constant rate and that the rate of administration of the IgG 10% should not be increased above the recommended rates, particularly when the patient has just started with HyQvia therapy.

First, the full dose of recombinant human hyaluronidase solution is infused at a rate of 1 to 2 ml/minute per infusion site. Within 10 minutes of completing the infusion of recombinant human hyaluronidase, the infusion of the required dose of IgG 10% has to be initiated at the same needle site. If two infusion sites are used, the total dosages of the recombinant human hyaluronidase and IgG 10% each have to be divided before start of the infusion.

The following infusion rates of the IgG 10% are recommended:

- Patients with a body weight of 40 kg or above: IgG 10% should be infused at an initial rate of 10 ml/hour/infusion site. If well tolerated, the rate of the administration may be increased at intervals of at least 10 minutes to a maximum of 240 ml/hour/site for the initial one or two infusions. For subsequent infusions the rate can be adjusted to a maximum of 300 ml/hour/site.

- Patients with a body weight under 40 kg: IgG 10% should be infused at an initial rate of 5 ml/hour/infusion site. If well tolerated, the rate of the administration may be increased at intervals of at least 10 minutes to a maximum of 80 ml/hour/site for the initial one or two infusions. For subsequent infusions the rate can be adjusted to a maximum of 160 ml/hour/site.

Patients will be advised and trained to perform the subcutaneous infusion by themselves at home.

3.7.6. Blinding

The study is open-label and unblinded.

3.7.7. Previous and concomitant medication

Patients are allowed to take additional medications. However, no new drugs for treatment of CFS are allowed while on study treatment with the exception of drugs, which are required to treat new symptoms requiring therapy.

The following medication is not allowed while on study treatment: Steroids in therapeutic dose (above 5 mg per day), any other immunomodulatory drugs.

3.7.8. Treatment after completion of clinical study

HyQvia (IgG 10%) is approved and marketed in Germany. After study completion the medication can be prescribed according to the labeled indication. Patients can stay on treatment, if the drug is efficacious and well tolerated.

3.8. Effectiveness and safety parameters

3.8.1. Measurement of effectiveness and safety parameters

3.8.1.1. Primary End Points

The Chalder Fatigue Scale and the SF-36 physical domain will be used for the assessment of the primary effectiveness endpoints.

- Chalder Fatigue Scale

The patient-rated primary endpoint measure of fatigue will be assessed with the Chalder Fatigue Scale at study inclusion and follow-up at 12 months. The Chalder Fatigue Scale is a self-administered questionnaire for measuring the extent and severity of both physical and psychological fatigue [10, 11]. Each of the 11 items are answered on a 4-point scale ranging from the asymptomatic to maximum symptomology, such as ‘Better than usual’, ‘No worse than usual’, ‘Worse than usual’ and ‘Much worse than usual’. Responses receive a score of

0, 1, 2 or 3 being the most severe. The global composite score can range from 0 to 33.

- SF-36 physical function

The Short Form (36) Health Survey is a 36-item, patient-reported survey of patient health [12, 13] [14]. The SF-36 consists of eight domains. The physical functioning section has 10 items. Each of the 10 items is answered on a 3-point scale ranging from maximum symptomology to asymptomatic, such as 'severly impaired', 'moderately impaired', 'not impaired''. Responses receive a score of 0, 5, or 10 with 0 being the most severe. The global composite score ranges from 0 to 100.

3.8.1.2. Secondary End Points

- The number of infections will be documented by patient's diary (type of infection, symptoms, duration, severity, antibiotics, hospitalization) to show effectiveness to control infections. This documentation includes the all infections 12 months prior to study enrollment and at each study visit for the last 3 months.
- Assessment of muscular fatigue, endothelial dysfunction and disturbed body composition as parameters of disease severity will be measured by arm and leg muscle power, composition of body tissues by bioelectrical impedance analysis (BIA), vessel function by flow mediated dilatation and activity tracking. These are established and validated diagnostic tests performed in the Charité Centrum für Schlaganfallforschung. A Viofit activity tracker from Garmin will be used to count daily activity including steps, hours of activity and hours of sleep for one week every 3 months.
- Additional questionnaires will be used to assess CFS symptoms and physical functioning:

  The CFS symptom scoring is adapted from Fluge et al. to asses and quantify symptoms of CFS based on the Canadian criteria [1, 15, 16]. Severity of symptoms will be assessed with a 10 point scale with 10 the most severe symptoms and Bell scale for disease severity [17].

The COMPASS-31 is a questionnaire validated by the Mayo Clinic to assess and quantify symptoms of autonomic dysfunction [18]. 6 categories of organ dysfunction will be assessed with a maximum of 100 being the most severe disturbance.

- An additional questionnaire (Infusionsbericht) will be used to assess IgG tolerability and handling of the mechanical pump.

3.8.1.3. Safety analysis

Patients are monitored for AE and SAE during IgG infusion as well as the periods between infusions. At home patients are requested to keep a diary where they document all infusions, complaints and problems. Side effects will be documented according to CTC criteria.

3.8.2. Appropriateness of methods

Assessment of infections and fatigue as described has been shown to be reliable already in many clinical trials.

The Chalder Fatigue Scale is a validated questionnaire to assess and quantify fatigue. The CFS Fatigue Score is adapted from Fluge et al. to assess and quantify symptoms of CFS (1). COMPASS-31 is a questionnaire validated by the MAYO Clinic to assess and quantify symptoms of autonomic dysfunction. SF-36 is a validated questionnaire to assess quality of live with a subdomaine for physical functioning. (Questionnaires attached in the Addendum, references [19-21] refer to clinical trials in CFS using these questionnaires).

3.8.3. Pharmacokinetic measurement

IgG serum trough level will be measured at baseline and every 3 months thereafter.

3.9. Monitoring

It is planned that during the course of the study, on-site monitoring with validation of the data of 4 of the 15 patients in the database against source data in the patient files will be performed. The study monitor is responsible for ensuring and verifying that each study site conducts the study according to the protocol, other written instructions/agreements and applicable regulatory guidelines/requirements. The investigator will permit the study monitor to visit the study site.

3.10. Documentation

The investigator or another adequately trained member of the study team will document all relevant data from patient visits in the paper case report form (CRF). The principal investigator has to sign the CRF pages to ascertain completeness and accuracy of the data. These criteria and unselected documentation of all results form an essential precondition for the scientific use of the collected data.

3.10.1. Data collection and pseudonymisation

The data will be entered on standardized case report (CRF) forms. The data will be entered manually by a trained person into a database (Microsoft ACCESS database). Data will be recorded pseudonymized, i.e., only the treating physician knows the identity of the patient.

3.10.2. Subject completion/ discontinuation

The primary reason for study discontinuation will be documented in the CRF in a separate section referring to the end-of-study. One of the following conditions should be met:

- Subject completed the study according to protocol
- Subject experienced adverse event(s) from HyQvia necessitating discontinuation of treatment
- Subject was withdrawn by the treating physician at the site for non-drug-related reasons

- Unsatisfactory therapeutic response to surveillance product (as determined by the treating physician)
- Subject voluntarily withdrew consent
- Subject was lost to follow-up
- Subject died
- Other reason for premature surveillance termination

Regardless of the reason for termination, all data available for the subject up to the time of termination should be recorded onto the appropriate CRF, prior to retrieval by the Sponsor. In the event of premature surveillance termination resulting from an adverse event, clinical and/or laboratory investigations that are beyond the scope of the required surveillance observations may be performed as part of the evaluation of the event at the discretion of the surveillance site treating physician. The treating physician shall provide follow-up information on subjects who experienced serious adverse events until a diagnosis and final outcome are established.

3.10.3. Archiving

All CRFs, signed patient informed consents and relevant study documents will be stored in accordance with §13 Abs. 10 GCP-V for at least 10 years.

4. Ethical and regulatory aspects

4.1. Independent ethical committee

Before initiation of the study, study materials (including protocol, case report form and patient information / consent form) will be submitted for ethical review at the Ethik-Kommission des Landes Berlin.

The study will only be started after the approval letters of the ethical committee (EC) and applicable regulatory authorities has been obtained. The EC's composition or a statement that the EC's composition meets applicable regulatory criteria will be documented.

If the protocol or any other information given to the subject is amended, the revised documents will be reviewed and approved/given favorable opinion by the EC and relevant regulatory authorities, where applicable. The protocol amendment will only be implemented upon the responsible party's receipt of approval and, if required, upon the responsible party's notification of applicable regulatory authority(ies) approval.

4.2. Ethical implementation

This study protocol and all possible amendments are written in accordance with the Declaration of Helsinki version October 1996 (48th General Assembly of the World Medical Association, Somerset West, Republic of South Africa).

4.2.1. Legal provisions and guidelines

This clinical study will be conducted in accordance with the principles of Good Clinical Practice (ICH-GCP) and the regulations of the actual Arzneimittelgesetz (AMG).

4.3. Approval and registration

Before study start, all documents will be submitted to the relevant authorities (Paul-Ehrlich-Institut (PEI) for approval.

4.4. Information and consent of study participants

Investigators will choose patients for enrollment considering the study eligibility criteria. The investigator will exercise no selectivity so that no bias is introduced from this source. A patient log will be kept, with a collection of information on non-included patients (with reasons for non-inclusion).

All patients must sign an informed consent form before entering into the study according to applicable regulatory requirements. Before use, the informed consent form will be reviewed by the responsible party and approved by the EC and regulatory authority(ies).

The informed consent form will include a comprehensive explanation of the study without any exculpatory statements, in accordance with the elements required by applicable regulatory requirements. Patients will be allowed sufficient time to consider participation in the study.

By signing the informed consent form, patients agree to participate in the study, unless they withdraw voluntarily or are terminated from the study for any reason.

The responsible party will provide to the investigator in written form any new information that significantly bears on the subjects' risks associated with medicinal product exposure. The informed consent will be updated, if necessary. This new information and/or revised informed consent form that has been approved by the applicable EC and regulatory authorities, where applicable, will be provided by the investigator to the subjects who consent to participate in the study.

4.5. Patient insurance

Patient insurance was procured by Charité with HD Gerling.

4.6. Data privacy

Data privacy law regulations will be followed. It is ensured that all material or data are only scientifically used according to data privacy regulations.

Study participants will be informed about the use of their pseudonymized data according to § 12 und § 13 GCP-V. If a patient does not consent he / she will not be included in the study.

5. Statistical methods and sample size calculation

5.1. Statistical analysis plan

This is an exploratory observational study. The type 1 error rate will not be adjusted in case of multiple comparisons. Multivariable statistical models will be used in the analyses of the primary and secondary study endpoints to analyse for a potential association with sex, infection onset, and presence of autoantibodies.

The intention-to-treat (ITT) analysis includes all patients receiving at least one dose of HyQvia. For patients who withdraw from the study or do drop-out for other reasons, the last available visit is used for analysis (last observation carried forward, LOCF). Other imputation methods will not be used. Further a per-protocol analysis will be performed on all patients who received the 12 months treatment as scheduled. Details on the analysis strategy will be described in the statistical analysis plan.

5.1.1. Description of patient sample

In order to obtain an unselected patient sample, all consecutive patients in the clinic who are eligible for participation and willing to participate will be included. The primary population for the analysis is the ITT LOCF population, which is defined as using only the last treatment assessment.

5.1.2. Analysis of the primary efficacy endpoint

The main analysis of the primary endpoint is the test for a significant improvement in the Chalder Fatigue Scale and the SF-36 physical functioning between pretreatment and 12 months follow-up. The change in the primary endpoint measures Chalder Fatigue Scale and the SF-36 physical functioning are analyzed between enrollment and 12-months follow-up. Generalized linear mixed models will be used for the analyses of the primary endpoints to model the individual change in these parameters during the study period. Therefore, it is possible to include all assessed follow-up visits into analysis. Furthermore, a second analyses aims to estimate the number of patients with a clinical important difference in the

CFS and SF-36 physical functioning between enrollment and 12-months follow-up. A clinical important improvement is defined by an improvement of at least 50% of symptoms in Chalder Fatigue Scale between the first visit and the 12-month follow-up visit. For this an improvement in at least 6 of the 11 items for minimum of one point improvement is required. It means that the composite score decreases by at least 6 points between enrollment and 12-months follow-up. For the SF-36 physical functioning, a clinically meaningful improvement is defined by an improvement of at least 50% of symptoms between study enrollment and the 12-month follow-up. A patient has a clinically meaningful improvement if the patient scores better in at least 5 of the 10 items for minimum at 12-months follow-up. It means that the composite score increases by at least 25 points between enrollment and 12-months follow-up.

To sum up, in order to verify the hypothesis of a possible effect of HyQvia treatment on the Chalder Fatigue Scale and SF-36, a decrease of at least 6 points in the Chalder Fatigue Scale or an increase of 25 points in the SF-36 physical functioning is required between enrollment and 12-months follow-up in the ITT LOCF population.

5.1.3. Analysis of the secondary efficacy endpoints

Observed changes from baseline to the 1-year observation for the ITT LOCF population will be calculated for all secondary endpoints as follows. Also, values at the visits at months 3, 6, and 9 will be presented descriptively (if available).

- CFS Symptom severity (max 280 points)
- Bell score (max 100 points)
- COMPASS-31 (abbreviated Composite Autonomic Symptom Score)
- Numbers of infections: comparison of the number in the 12 months prior to study onset and in the 12 months during study treatment.
- Muscle strength (as measure of functionality)
- composition of body tissues by bioimpedance,
- vessel function by flow mediated dilatation
- Patient satisfaction rates with IgG and pumps, by questionnaires at months 6

- activity tracker: steps and hours of activity per day

The change in the secondary endpoint measures is analyzed between enrollment and 12-months follow-up. Generalized linear mixed models will be used for the analyses of the secondary endpoints to model the individual change in these parameters during the study period. Therefore, it is possible to include all assessed follow-up visits into analysis.

5.1.4. Subgroup analyses

Due to the small sample size, no subgroup analyses are planned.

5.1.5. Interim analyses

Not planned.

5.2. Sample size calculation

Given this is a proof-of-concept study, no formal sample size calculation has been performed. Sample size is determined using feasibility considerations.

6. Documentation of adverse events and adverse reactions

Adverse events should be documented in the patients file and the CRF noting the following parameters:

- date and time of event start and end
- intensity
- causality
- serious or non-serious
- discontinuation or stopping of study drug and other taken actions

6.1. Adverse event

An AE is defined as any untoward medical occurrence in a subject administered medicinal product that does not necessarily have a causal relationship with the treatment. An AE can therefore be any unfavorable and unintended sign (e.g., an abnormal laboratory finding), symptom (e.g., rash, pain, discomfort, fever, dizziness, etc.), disease (e.g., peritonitis, bacteremia, etc.), or outcome of death temporally associated with the use of a medicinal product, whether or not considered causally related to the medicinal product. Events that do not necessarily meet the definition of AEs, regardless of causal association with medicinal product, should be treated as AEs because they may be reportable to Regulatory Authorities according to AE reporting regulation; these include the following:

- Medicinal product overdose, whether accidental or intentional
- Medicinal product abuse
- An event occurring from medicinal product withdrawal
- Any failure of expected pharmacological action
- Exposure to medicinal product during pregnancy
- Unexpected therapeutic or clinical benefit from the medicinal product

Each AE from the first medicinal product exposure until study completion will be described on the AE CRF using the medical diagnosis (preferred), or, if no diagnosis could be established at the time of reporting the AE, a symptom or sign, in standard medical terminology in order to avoid the use of vague, ambiguous, or colloquial expressions Each AE will be evaluated by the investigator for:

For each AE, the outcome (i.e., recovering/resolving, recovered/resolved, recovered/resolved with sequelae, not recovered/not resolved, fatal, unknown) and action taken (i.e., dose increased, dose not changed, dose reduced, drug interrupted, drug withdrawn, not applicable, or unknown) will also be recorded on the AE CRF. Recovering/resolving AEs will be followed until resolution, medically stabilized, or 30 days after the study completion/termination, whichever comes first.

If an investigator becomes aware of an SAE occurring in a subject after study completion, the

preexisting diseases that are present before entry in to the study are described in the medical history; those that manifest with the same severity, frequency, or duration after medicinal product exposure, will not be recorded as AEs. However, when there is an increase in the severity, duration, or frequency of a preexisting disease, the event must be described on the AE CRF.

Preexisting diseases

Preexisting diseases that are present before entry in to the study, described in the medical history, and that manifest with the same severity, frequency, or duration after study medication exposure, will not be recorded as AEs. However, when there is an increase in the severity or duration of a preexisting disease, the event must be described on the AE CRF.

Pregnancy

If a woman becomes pregnant, the treating physician should encourage her to participate in the pregnancy registry that is described in Baxalta Protocol 161301: Registry Study to collect Long-Term Safety Data from Female Subjects who become pregnant during treatment with HyQvia (Immune Globulin (Human) 10% with rHuPH20).

6.2. Adverse Drug Reaction

Adverse drug reaction (ADR) is every unintended adverse reaction to the study medication (in this study HyQvia) independent of dose and with an at least possible causality with the application of the study medication.

6.3. Serious Adverse Event

A serious adverse event (SAE) is defined as an untoward medical occurrence that at any dose meets one or more of the following criteria:

- Outcome is fatal/results in death (including fetal death)
- Is life-threatening – defined as an event in which the subject was, in the judgment of the investigator, at risk of death at the time of the event; it does not refer to an event that hypothetically might have caused death had it been more severe.
- Requires inpatient hospitalization or results in prolongation of an existing hospitalization – inpatient hospitalization refers to any inpatient admission, regardless of length of stay.
- Results in persistent or significant disability/incapacity (i.e., a substantial disruption of a person’s ability to conduct normal life functions)
- Is a congenital anomaly/birth defect
- Is a medically important event – a medical event that may not be immediately life-threatening or result in death or require hospitalization but may jeopardize the subject or may require medical or surgical intervention to prevent one of the other outcomes listed in the definitions above. Examples of such events are:
  - Intensive treatment in an emergency room or at home for allergic bronchospasm, blood dyscrasias, or convulsions that do not result in hospitalization, or development of drug dependence or drug abuse
  - Thromboembolic events (e.g., deep vein thrombosis, pulmonary embolism, myocardial infarction, transient ischaemic attack, stroke, etc.)

- Diagnosis of hemolytic anemia, reviewed and confirmed by the using standard laboratory assessments

6.4. Unexpected Adverse Events

An unexpected adverse event is an AE whose nature, severity, specificity, or outcome is not consistent with the term, representation, or description used in the Reference Safety Information (e.g., product labeling, SmPc). “Unexpected” also refers to the AEs that are mentioned in the product labeling as occurring with a class of medicinal products or as anticipated from the pharmacological properties of the medicinal product, but are not specifically mentioned as occurring with the particular medicinal product under investigation.

6.5. Suspected Unexpected Adverse Reaction

A Suspected Unexpected Adverse Reaction (SUSAR) is an adverse event that is, regarding kind and severity, not in accordance with the effective SmPc of the study medication. It should be severe and causality should at least be possible.

6.6. Documentation and follow up of adverse events

The Sponsor is responsible that all persons involved in the treatment of the study patients are aware of their responsibility in handling adverse events. At every visit patients have to be interviewed if unexpected or severe adverse events have occurred. Adverse events should be documented in the patients file and the CRF (see also 7.3.).

6.7. AE Severity

The investigator will assess the severity of each AE using his/her clinical expertise and judgment based on the most appropriate description below:

- Mild

  The AE is a transient discomfort and does not interfere in a significant manner with the subject’s normal functioning level.

The AE resolves spontaneously or may require minimal therapeutic intervention.

- Moderate

  The AE produces limited impairment of function and may require therapeutic intervention.

  The AE produces no sequel.

- Severe

  The AE results in a marked impairment of function and may lead to temporary inability to resume usual life pattern.

  The AE produces sequel, which require (prolonged) therapeutic intervention.

6.8. Causality of adverse event

Causality is a determination of whether there is a reasonable possibility that the medicinal product is etiologically related to/associated with the AE. Causality assessment includes, e.g., assessment of temporal relationships, dechallenge/ rechallenge information, association (or lack of association) with underlying disease, presence (or absence) of a more likely cause, and physiological plausibility. For each AE assessed as not related or unlikely related, the investigator shall provide an alternative etiology. For each AE, the investigator will assess the causal relationship between the medicinal product and the AE using his/her clinical expertise and judgment according to the following most appropriate algorithm for the circumstances of the AE (WHO Causality Assessment of Suspected Adverse Reactions):

Certain:

- Event or laboratory test abnormality, with plausible time relationship to drug intake
- Cannot be explained by disease or other drugs
- Response to withdrawal plausible (pharmacologically, pathologically)
- Event definitive pharmacologically or phenomenologically (i.e. an objective and specific medical disorder or a recognized pharmacological phenomenon)

- Rechallenge satisfactory, if necessary

Probable/Likely:

- Event or laboratory test abnormality, with reasonable time relationship to drug intake
- Unlikely to be attributed to disease or other drugs
- Response to withdrawal clinically reasonable
- Rechallenge not required

Possible:

- Event or laboratory test abnormality, with reasonable time relationship to drug intake
- Could also be explained by disease or other drugs
- Information on drug withdrawal may be lacking or unclear

Unlikely:

- Event or laboratory test abnormality, with a time to drug intake that makes a relationship improbable (but not impossible)
- Disease or other drugs provide plausible explanations

Conditional/Unclassified:

- Event or laboratory test abnormality
- More data for proper assessment needed, or
- Additional data under examination

Unassessable/Unclassifiable:

- Report suggesting an adverse reaction
- Cannot be judged because information is insufficient or contradictory

- Data cannot be supplemented or verified

6.9. Reporting of serious adverse event, and pregnancies

In accordance with applicable regulatory requirements all serious adverse event (independent of causality will be reported to the “Bundeoberbehörde” (PEI) and the Independent ethical committee (EC).

Further serious adverse event will be reported within 24 hours of awareness to the pharmacovigilance department of Baxalta (marketing authorization holder of the study drug HyQvia):

global_pharmacovigilance_deerfield@baxalta.com

vigilance_germany@baxalta.com

In addition, all pregnancies will be reported to the EMA HyQvia pregnancy registry (“Registry Study to collect Long-Term Safety Data from Female Subjects who become pregnant during treatment with HyQvia (Immune Globulin (Human) 10% with rHuPH20”).

7. Data utilisation and publication

7.1. Study reports

The Bundesoberbehörde (PEI) and the independent ethical committee will be notified within 90 days about the completion of the study.

Within one year after study completion the Bundesoberbehörde (PEI) and the independent ethical committee will receive a summary of the final report with all relevant study results.

7.2. Publication

It is planned to publish the results of the clinical study in a peer reviewed journal.

Publication recommendations according to „Uniform requirements for manuscripts submitted to biomedical journals (International Committee of Medical Journal Editors" (ICMJE) will be followed.

8. Changes to the study protocol

All subsequent chances to the protocol have to be submitted and approved by the Bundesoberbehörde (PEI) and the independent ethical committee, with the exemption of chances to prevent immediate danger.

9. References

[1] Fluge O, Risa K, Lunde S, Alme K, Rekeland IG, Sapkota D, et al. B-Lymphocyte Depletion in Myalgic Encephalopathy/ Chronic Fatigue Syndrome. An Open-Label Phase II Study with Rituximab Maintenance Treatment. PloS one. 2015;10:e0129898.
[2] Loebel M, Grabowski P, Heidecke H, Bauer S, Hanitsch LG, Wittke K, et al. Antibodies to beta adrenergic and muscarinic cholinergic receptors in patients with Chronic Fatigue Syndrome. Brain, behavior, and immunity. 2016;52:32-9.
[3] Guenther S, Loebel M, Mooslechner AA, Knops M, Hanitsch LG, Grabowski P, et al. Frequent IgG subclass and mannose binding lectin deficiency in patients with chronic fatigue syndrome. Human immunology. 2015;76:729-35.
[4] Loebel M, Mooslechner AA, Bauer S, Guenther S, Letsch A, Hanitsch LG, Grabowski P, Meisel C, Volk HD and Scheibenbogen S Polymorphism in COMT is associated with IgG3 subclass level and susceptibility to infection in patients with Chronic Fatigue Syndrome. Journal of translational medicine. 2015;in press.
[5] Whiting P, Bagnall AM, Sowden AJ, Cornell JE, Mulrow CD, Ramirez G. Interventions for the treatment and management of chronic fatigue syndrome: a systematic review. Jama. 2001;286:1360-8.
[6] Smith ME, Haney E, McDonagh M, Pappas M, Daeges M, Wasson N, et al. Treatment of Myalgic Encephalomyelitis/Chronic Fatigue Syndrome: A Systematic Review for a National Institutes of Health Pathways to Prevention Workshop. Annals of internal medicine. 2015;162:841-50.
[7] Wasserman RL, Melamed I, Stein MR, Gupta S, Puck J, Engl W, et al. Recombinant human hyaluronidase-facilitated subcutaneous infusion of human immunoglobulins for primary immunodeficiency. The Journal of allergy and clinical immunology. 2012;130:951-7.e11.
[8] Baxalta. HyQvia Fachinformation. 2016 Juni.
[9] Sanford M. Human immunoglobulin 10 % with recombinant human hyaluronidase: replacement therapy in patients with primary immunodeficiency disorders. BioDrugs : clinical immunotherapeutics, biopharmaceuticals and gene therapy. 2014;28:411-20.
[10] Chalder T, Berelowitz G, Pawlikowska T, Watts L, Wessely S, Wright D, et al. Development of a fatigue scale. Journal of psychosomatic research. 1993;37:147-53.
[11] Jackson C. The Chalder Fatigue Scale (CFQ 11). Occupational medicine (Oxford, England). 2015;65:86.
[12] Ware JE, Jr., Sherbourne CD. The MOS 36-item short-form health survey (SF-36). I. Conceptual framework and item selection. Medical care. 1992;30:473-83.
[13] Hays RD, Shapiro MF. An overview of generic health-related quality of life measures for HIV research. Quality of life research : an international journal of quality of life aspects of treatment, care and rehabilitation. 1992;1:91-7.
[14] Stewart AL SC, Hays RD et al. . Summary anbd Discussion of MOS Measures: Durham NC, Duke University Press; 1992.
[15] Carruthers BM, van de Sande MI, De Meirleir KL, Klimas NG, Broderick G, Mitchell T, et al. Myalgic encephalomyelitis: International Consensus Criteria. Journal of internal medicine. 2011;270:327-38.
[16] Carruthers BM JA, De Meirleir KL. Myalgic encephalomyelitis/chronic fatigue syndrome: clinical working case definition, diagnostic and treatment protocols. J Chronic Fatigue Syndr 2003:7–116.

[17] Bell DS. The Doctor's Guide to Chronic Fatigue Syndrome: Addison-Wesley, Publishing Company, Reading.
[18] Sletten DM, Suarez GA, Low PA, Mandrekar J, Singer W. COMPASS 31: a refined and abbreviated Composite Autonomic Symptom Score. Mayo Clinic proceedings. 2012;87:1196-201.
[19] Sharpe M, Goldsmith KA, Johnson AL, Chalder T, Walker J, White PD. Rehabilitative treatments for chronic fatigue syndrome: long-term follow-up from the PACE trial. The lancet Psychiatry. 2015;2:1067-74.
[20] McCrone P, Sharpe M, Chalder T, Knapp M, Johnson AL, Goldsmith KA, et al. Adaptive pacing, cognitive behaviour therapy, graded exercise, and specialist medical care for chronic fatigue syndrome: a cost-effectiveness analysis. PloS one. 2012;7:e40808.
[21] Jason L, Benton M, Torres-Harding S, Muldowney K. The impact of energy modulation on physical functioning and fatigue severity among patients with ME/CFS. Patient education and counseling. 2009;77:237-41.

Appendix Questionnaires <u>and CRF</u>

- Chalder Fatigue Scale
- SF-36
- CFS symptom score
- Bell scale
- Canadian criteria
- COMPASS-31
- Infusionsbericht
- CFR
