# Supplementary material for: Tolerability and Efficacy of s.c. IgG Self-Treatment in ME/CFS Patients with IgG/IgG Subclass Deficiency: A Proof-of-Concept Study
Source: J Clin Med. 2021 May 29;10(11):2420. doi: 10.3390/jcm10112420 (PMC8198960; doi:10.3390/jcm10112420)
Supplement: Supplementary file 1 [file jcm-10-02420-s001.zip › Supplementary Tables and Figures.pdf]

Supplementary Tables and Figures

Tolerability and efficacy of s.c. IgG self-treatment in patients with ME/CFS: a proof-of-concept study

Carmen Scheibenbogen[*1,2], Franziska Sotzny[1], Jelka Hartwig[1], Sandra Bauer[1], Helma Freitag[1], Kirsten Wittke[1], Wolfram Doehner[2,3], Nadja Scherbakov[2,3], Madlen Loebel[4], Patricia Grabowski[1]

Journal of Clinical Medicine, 2021

[*] **Corresponding author:**

Carmen Scheibenbogen
Institute of Medical Immunology, Charité – Universitätsmedizin Berlin, corporate member of Freie Universität Berlin and Humboldt-Universität zu Berlin, Germany

Carmen.Scheibenbogen@charite.de

Table S1: Patient characteristics and treatment response

Pat.	sex/ age	age at disease onset	Bell	infection triggered onset	IgG deficiency	treatment prematurely ceased	responder month 12	response at month 6 or 9
1	m/44	30	50	no	IgG	2 injections		
2	m/33	17	40	yes EBV	IgG4		yes	
3	w/54	45	25	no	IgG3	month 6		
4	w/39	36	40	yes, respiratory tract infection	IgG/IgG1			yes
5	m/37	28	20	yes EBV	IgG2			
6	m/60	55	30	no	IgG3			
7	w/32	31	30	yes, respiratory tract infection	IgG3			
8	w/70	42	30	yes Herpes zoster	IgG			yes
9	m/46	43	30	yes borrilia	IgG3		yes	
10	w/62	61	40	yes peri-myocarditis	IgG			
11	w/49	47	20	yes EBV	IgG3		yes	
12	m/18	15	20	no	IgG4	month 3		
13	w/38	36	20	yes respiratory tract infection	IgG3		yes	
14	m/29	28	40	yes bronchitis	IgG4	1 injection		
15	w/54	51	45	yes respiratory tract infection	IgG3/IgG4	month 3		
16	w/46	36	20	yes *Mycoplasma pneumoniae*	IgG4		yes	
17	m/54	42	30	no	IgG			

Table S2: Adverse events

adverse event	treatment completed (n=12)	treatment not completed (n=5) (P1, P3, P12, P14, P15)
injection site reaction	grade 1: n=10<br>grade 2: n=2 (P13, P11)	grade 1: n=3<br>grade 2: n=2 (P3, P14)
flu-like symptoms	grade 1: n=11<br>grade 2: n=1 (P4)	grade 1: n=2<br>grade 2: n=1 (P3)
headache	grade 1: n=2<br>grade 2: n=3 (P4, P7, P10)	grade 1: n= 3<br>grade 2: n=1 (P3)<br>grade 3: n=1 (P1)
abdominal pain	grade 2: n=1 (P4)	grade 1: n=3<br>grade 2: n=1 (P3)
diarrhea	grade 1: n=2	grade 1: n=2
liver toxicity (ALT/GPT increase)	grade 1: n=3	grade 3 n=1 (P15)

Grade refers to the severity of the adverse event, as described in the "Common Terminology Criteria for Adverse Events (CTCAE)" Version 5. (grade 1: mild, grade 2: moderate, grade 3: severe)

Table S3: Laboratory values pretreatment (median with interquartile range [IQR])

parameter	responder at month 12 (n=5)	non-responder (n=7)	reference range
leukocytes [/nl]	7.96 (4.86/8.59), n=5	6.31 (5.86/8.66), n=7	3.90-10.50
lymphocytes [/nl]	1.86 (1.635/2.565), n=5	2.07 (1.705/2.425), n=7	1.50-3.00
erythrocytes [/pl]	4.5 (4.45/5.45), n=5	4.9 (4.5/5.2), n=7	4.3-5.8
Hb [g/dl]	14 (12.8/16.35), n=5	14.8 (13.7/15.1), n=7	13.5-17.0
MCV [fl]	86 (80.5/87), n=5	85 (83/87), n=7	80.0-99.0
MCHC [g/dl]	35.8 (34.15/36.15), n=5	35 (34/35.7), n=7	31.5-36.0
LDH [U/l]	298 (242.5/314), n=5	225 (212/266), n=7	135-250
CK [U/l]	121 (52.5/192.5), n=5	89 (63.75/113.5), n=6	< 190
CRP [mg/dl]	0.7 (0.4/2.05), n=5	0.8 (0.3/3.6), n=7	< 5
IgG [g/l]	8.96 (7.825/9.925), n=5	6.55 (5.68/9.97), n=7	7.00-16.00
IgG1 [g/l]	3.923 (3.768/4.907), n=4	3.28 (3.043/6.315), n=7	2.800-8.000
IgG2 [g/l]	4.122 (2.514/4.644), n=5	2.510 (1.832/3.353), n=7	1.120-5.700
IgG3 [g/l]	0.282 (0.166/0.8255), n=5	0.376 (0.127/0.558), n=7	0.240-1.250
IgG4 [g/l]	0.12 (0.03/0.5195), n=5	0.138 (0.1/0.273), n=7	0.052-1.250
soluble IL-2 receptor [U/ml]	328 (259/367), n=5	475.5 (316.8/650.3), n=6	< 710
IL-8	126.6 (75.2/205.1), n=5	84.4 (70.75/145.1), n=6	< 150
ANA	negative	negative	n.a.
β2 AdR AAB [Units/ml]	4.83 (3.705/8.79), n=5	5.395 (2.638/7.84), n=6	n.a.
M3 AchR AAB [Units/ml]	3.49 (2.795/7.955), n=5	3.02 (2.188/4.115), n=6	n.a.

sCD26	866.5 (811.1/1058), n=5	722.6 (621.3/1061), n=7	n.a.

[ANA= Antinuclear antibodies; CK= creatine kinase; β2 AdR AAB= autoantibody against β2 adrenergic receptor; CRP= C-reactive protein; Hb= hemoglobin;Ig=immunoglobulin; IL=interleukin; LDH= lactate dehydrogenase; MCHC= mean corpuscular haemoglobin concentration; MCV= mean corpuscular volume; M3 AchR AAB= autoantibody against Muscarinic acetylcholine receptor M3; sCD26= soluble Dipeptidyl peptidase-4]

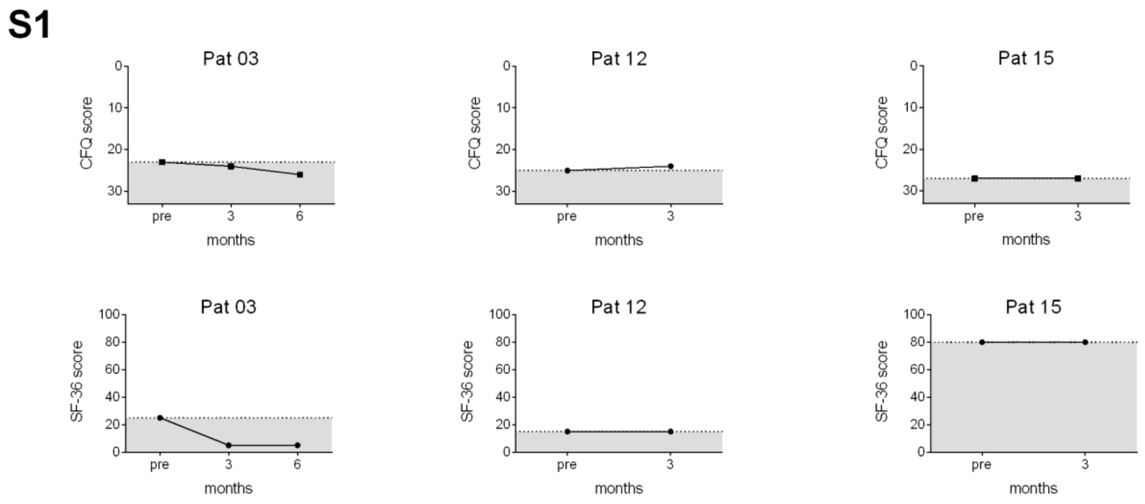

Figure S1: CFQ and the SF-36 data of patients 3 and 6.

S2

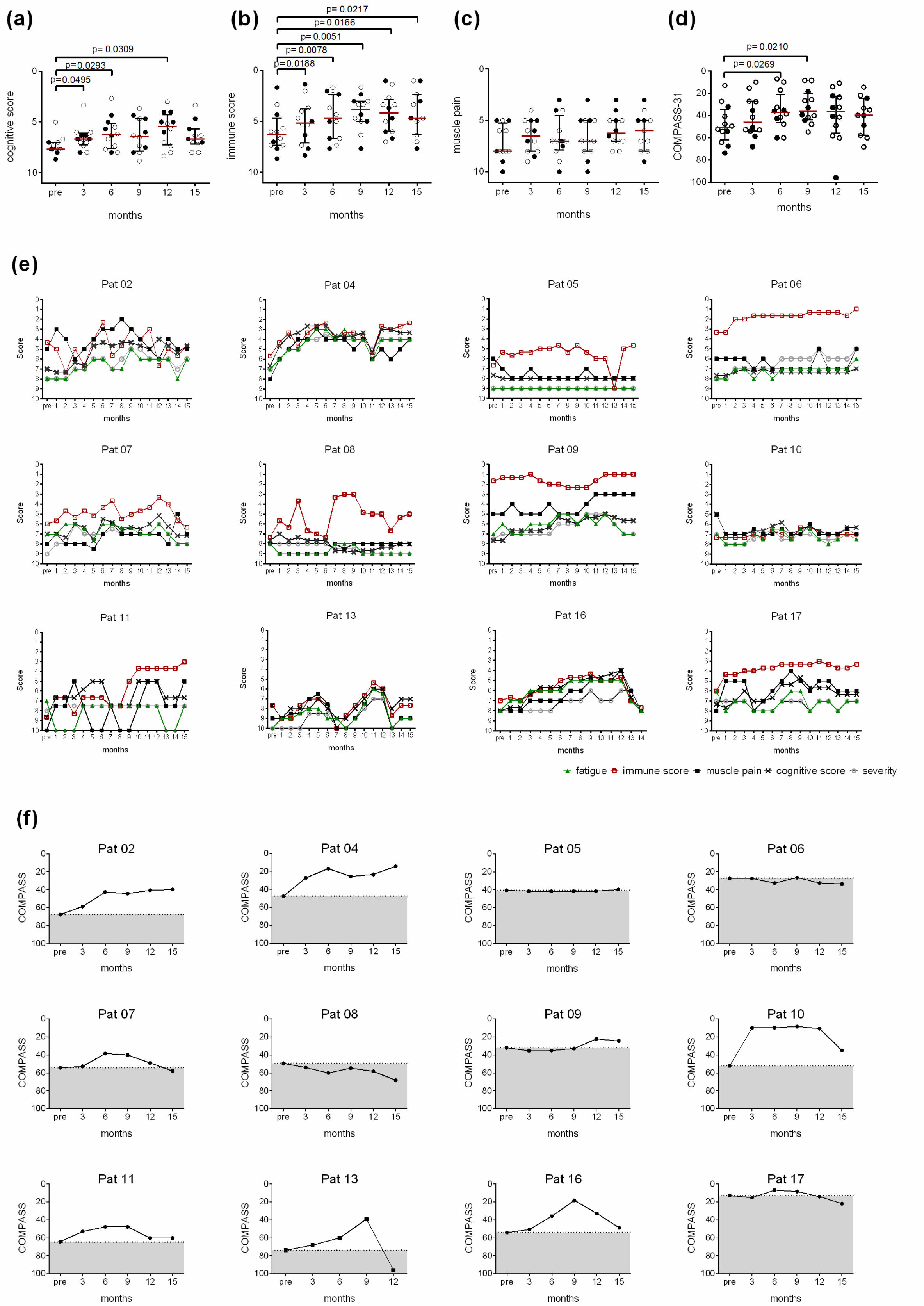
(a)
p= 0.0309
p=0.0293
p=0.0495
cognitive score
months
(b)
p= 0.0217
p= 0.0166
p= 0.0051
p= 0.0078
p= 0.0188
immune score
months
(c)
muscle pain
months
(d)
p= 0.0210
p= 0.0269
COMPASS-31
months
(e)
Pat 02
Pat 04
Pat 05
Pat 06
Pat 07
Pat 08
Pat 09
Pat 10
Pat 11
Pat 13
Pat 16
Pat 17
Score
months
fatigue
immune score
muscle pain
cognitive score
severity
(f)
Pat 02
Pat 04
Pat 05
Pat 06
Pat 07
Pat 08
Pat 09
Pat 10
Pat 11
Pat 13
Pat 16
Pat 17
COMPASS
months

Figure S2: Symptoms of patients during s.c. IgG therapy. Median with interquartile range of **(a-c)** CCC symptom scores and **(d)** autonomous dysfunction (COMPASS-31) of the patients before (pre), during (months 3-12) and 3 months after treatment (month 15) are shown (responder indicated as filled circles). An overall significant improvement of symptoms during the IgG treatment at various time points was observed. Two-tailed Wilcoxon matched-pairs signed-rank test was performed for statistical analysis. **(e)** CCC symptom scores and **(f)** COMPASS-31 of the patients before (pre), during (months 3-12) and 3 months after the treatment (month 15) are shown. (COMPASS Score, healthy: 0)

S3

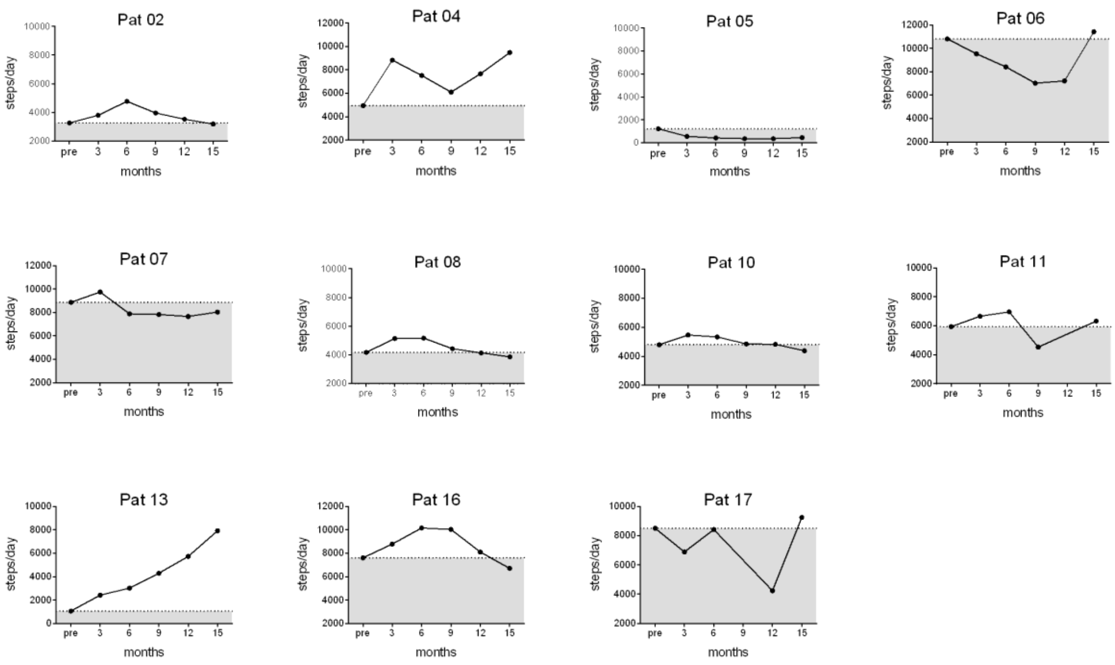

Figure S3: Daily steps assessed by Activity tracking. Steps of patients were counted by Vivofit® activity tracker during s.c. IgG therapy. Mean daily number of steps counted during one week before and thereafter monthly in 11 patients receiving 12 months of treatment.

S4

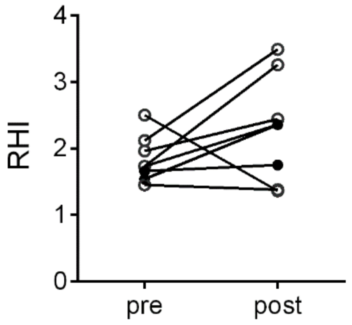

Figure S4: The reactive hyperemia index (RHI) assessed by Endopat. RHI of patients before (pre) and 3 months after the IgG treatment at month 15 (post) is plotted (responder indicated as filled circles).

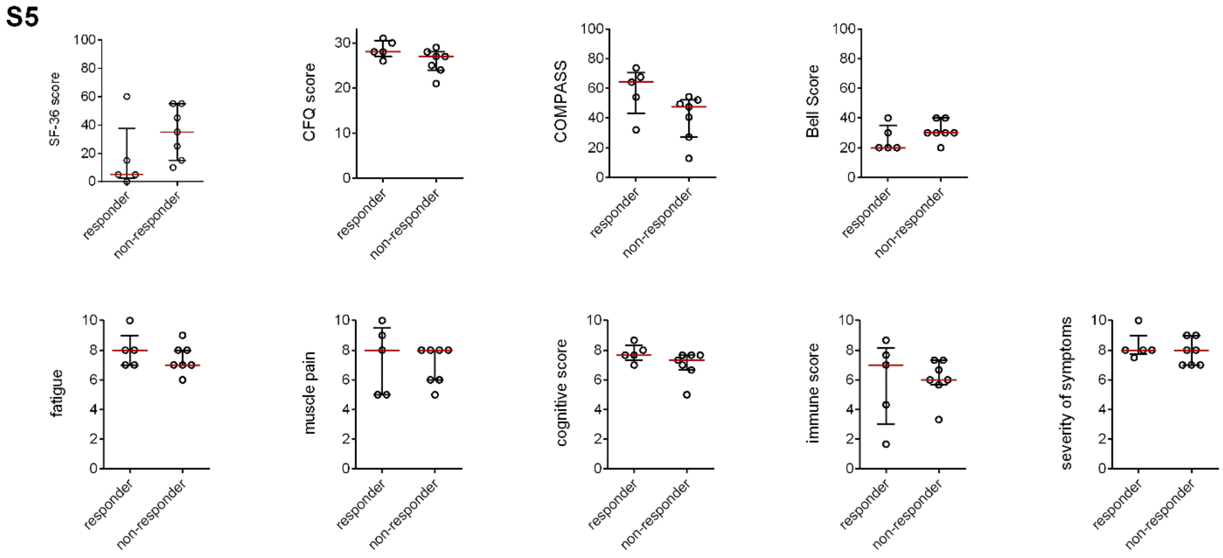

Figure S5: Comparative analysis of pretreatment symptoms of responder vs. non- responder. Median with interquartile ranges of SF-36, CFQ, COMPASS, Bell and CCC symptom scores of 5 responder and 7 non-responder patients, receiving 12 months of treatment, are shown.
